# Supplementary material for: Ameliorative Effect of Ethanolic Echinacea purpurea against Hyperthyroidism-Induced Oxidative Stress via AMRK and PPAR Signal Pathway Using Transcriptomics and Network Pharmacology Analysis
Source: Int J Mol Sci. 2022 Dec 22;24(1):187. doi: 10.3390/ijms24010187 (PMC9820381; doi:10.3390/ijms24010187)
Supplement: Supplementary file 1 [file ijms-24-00187-s001.zip › ijms-2078608-supplementary.pdf]

Supplementary Data

Table S1. Primers used for quantitative RT-qPCR

Table S2. Compounds in EEP by UPLC-Q-TOF-MS

ARRIVE CHECKLIST

Table S1. Primers used for quantitative RT-qPCR.

Genes	Forward (5'–3')	Reverse (5'–3')
GAPDH	CCTCGTCCCGTAGACAAAATG	TGAGGTCAATGAAGGGGTCGT
EHHADH	GTGGCTCTTGGAGGAGGACTA	TTCAACTGGGTCTGACTTTACAAC
SLC27A2	ATCTGGCTGGGACTGCTCAAAC	CGCCGACACTCCGTCTACTTT
ACOT8	CTCTTTGGGGGTCAAATTATGG	ACGGAGGGCATGGAGAACTG
EPHX2	ATGGCAGCAAGAAGCATCAAC	TGCCTTCAGGGTATCCAGTAAA
ACACA	AGGAAGTTGGCTATCCAGTGATG	AGCAGTCACGACCAAACAAAGA
ACOT2	GACCCTTTCCTGGGATCATAGAC	GCTTCTTCAAAGTACTCCATGTGC
FASN	TGAATCAGCCCCACGCAGT	CCGAGTCAGTCTTGGAGGACAT
ACSL4	ATGAATGTCTGCTTCTGCTGCC	ACCTCTGGGGTTCGGCTTAT
ACADM	AGAAGTATTTGGGGAGGATGACG	CCGTTGGTTATCCACATCTTCTG
HMGCR	GAATGCCTTGTGATTGGAGTTG	CATCTTGACCCTTTGGGTTACG

Table S2. Compounds in EEP by UPLC-Q-TOF-MS

Num	Time (min)	Adduct Ion	*m/z* value	*m/z* theoretical value	ppm	Formula	Molecula Weight	Name	MS/MS Data
1	4.69	$[M-H]^-$	311.0412	311.0409	0.3	$C_{13}H_{12}O_9$	312.05	Caftaric acid	179.0344;149.0085;135.0446
2	7.46	$[M-H]^-$	295.0460	295.0459	0.1	$C_{13}H_{12}O_8$	296.05	Coutaric acid	163.0394;119.0496;87.0078
3	9.66	$[M-H]^-$	325.0573	325.0565	0.8	$C_{14}H_{14}O_9$	326.06	Cis-fertaric acid	193.0501;149.0599;124.0348
4	10.18	$[M+FA-H]^-$	395.1920	395.1923	-0.3	$C_{16}H_{30}O_8$	350.19	Rhodioloside D	349.1851;187.1325;161.0442;119.0347;89.0238
5	11.92	$[M-H]^-$	325.0563	325.0565	-0.2	$C_{14}H_{14}O_9$	326.06	Trans-fertaric acid	193.0502;149.0597;124.0348
6	17.87	$[M-H]^-$	245.0934	245.0932	0.2	$C_{13}H_{14}N_2O_3$	246.10	N-Acetyl-DL-tryptophan	203.0815;159.0916;142.0651;116.0499;98.0241
7	19.31	$[M-H]^-$	473.0737	473.0725	1.2	$C_{22}H_{18}O_{12}$	474.08	Chicoric acid	293.0299;249.0371;219.0290;179.0353;149.0094
8	21.78	$[M-H]^-$	609.1461	609.1461	0.0	$C_{27}H_{30}O_{16}$	610.15	Quercetin 3-O-robinobioside	609.1457;301.0343;300.0268
9	22.43	$[M-H]^-$	609.1464	609.1461	0.3	$C_{27}H_{30}O_{16}$	610.15	Rutin	609.1460;301.0346;300.0268
10	22.85	$[M-H]^-$	463.0880	463.0882	-0.2	$C_{21}H_{20}O_{12}$	464.10	Isoquercitrin	463.0881;301.0338;300.0268
11	43.37	$[M-H]^-$	329.2341	329.2333	0.8	$C_{18}H_{34}O_5$	330.24	Trihydroxyoctadecanoic acid	329.2327;311.2214;229.1440.211.1334;
12	47.48	$[M+H]^+$	230.1554	230.1539	1.4	$C_{15}H_{19}NO$	229.15	2,4-Undecadiene-8,10-diynoic acid isobutylamide	230.1496;167.1262;152.1033;128.0582;91.0508
13	55.81	$[M+H]^+$	248.2025	248.2009	1.7	$C_{16}H_{25}NO$	247.19	Dodeca-2E,4E,8Z,10E-tetraenoic acid isobutylamide	248.1990;167.1287;152.1052
14	56.25	$[M+H]^+$	248.2023	248.2009	1.4	$C_{16}H_{25}NO$	247.19	Dodeca-2E,4E,8Z,10Z-tetraenoic acid isobutylamide	248.1963;167.1267;152.1034
15	74.86	$[M-H]^-$	271.2283	271.2279	0.4	$C_{16}H_{32}O_3$	272.24	Hydroxyhexadecanoic acid	271.2270;253.2145;225.2219
16	75.98	$[M-H]^-$	277.2176	277.2173	0.2	$C_{18}H_{30}O_2$	278.22	Linolenic acid	277.2170;259.2049;233.2256
17	78.83	$[M-H]^-$	279.2330	279.2330	0.0	$C_{18}H_{32}O_2$	280.24	Linoleic acid	279.2322;261.2216;211.1217;134.8927
18	81.44	$[M-H]^-$	255.2328	255.2330	-0.1	$C_{16}H_{32}O_2$	256.24	Palmitic acid	255.2320;241.5923;203.8309

ARRIVE CHECKLIST

NOTE: Please save this file locally before filling in the table, DO NOT work on the file within your internet browser as changes will not be saved. Adobe Acrobat Reader (available free here) is recommended for completion.

The ARRIVE guidelines 2.0: author checklist

The ARRIVE Essential 10

These items are the basic minimum to include in a manuscript. Without this information, readers and reviewers cannot assess the reliability of the findings.

Item		Recommendation	Section/line number, or reason for not reporting
**Study design**	1	For each experiment, provide brief details of study design including:	
		a. The groups being compared, including control groups. If no control group has been used, the rationale should be stated.	Section 4.4
		b. The experimental unit (e.g. a single animal, litter, or cage of animals).	Section 4.4
**Sample size**	2	a. Specify the exact number of experimental units allocated to each group, and the total number in each experiment. Also indicate the total number of animals used.	Section 4.4
		b. Explain how the sample size was decided. Provide details of any *a priori* sample size calculation, if done.	Section 4.4
**Inclusion and exclusion criteria**	3	a. Describe any criteria used for including and excluding animals (or experimental units) during the experiment, and data points during the analysis. Specify if these criteria were established *a priori*. If no criteria were set, state this explicitly.	Section 1.
		b. For each experimental group, report any animals, experimental units or data points not included in the analysis and explain why. If there were no exclusions, state so.	Section 2.
		c. For each analysis, report the exact value of *n* in each experimental group.	Section 4.4/2.
**Randomisation**	4	a. State whether randomisation was used to allocate experimental units to control and treatment groups. If done, provide the method used to generate the randomisation sequence.	Section 4.4
		b. Describe the strategy used to minimise potential confounders such as the order of treatments and measurements, or animal/cage location. If confounders were not controlled, state this explicitly.	Section 4.4
**Blinding**	5	Describe who was aware of the group allocation at the different stages of the experiment (during the allocation, the conduct of the experiment, the outcome assessment, and the data analysis).	Section 4.4
**Outcome measures**	6	a. Clearly define all outcome measures assessed (e.g. cell death, molecular markers, or behavioural changes).	Section 2.
		b. For hypothesis-testing studies, specify the primary outcome measure, i.e. the outcome measure that was used to determine the sample size.	Section 2.
**Statistical methods**	7	a. Provide details of the statistical methods used for each analysis, including software used.	Section 4.9
		b. Describe any methods used to assess whether the data met the assumptions of the statistical approach, and what was done if the assumptions were not met.	Section 4.9
**Experimental animals**	8	a. Provide species-appropriate details of the animals used, including species, strain and substrain, sex, age or developmental stage, and, if relevant, weight.	Section 4.
		b. Provide further relevant information on the provenance of animals, health/immune status, genetic modification status, genotype, and any previous procedures.	Section 4.
**Experimental procedures**	9	For each experimental group, including controls, describe the procedures in enough detail to allow others to replicate them, including:	
		a. What was done, how it was done and what was used.	Section 4.4
		b. When and how often.	Section 4.4
		c. Where (including detail of any acclimatisation periods).	Section 4.4
		d. Why (provide rationale for procedures).	Section 4.4
**Results**	10	For each experiment conducted, including independent replications, report:	
		a. Summary/descriptive statistics for each experimental group, with a measure of variability where applicable (e.g. mean and SD, or median and range).	Section 2.
		b. If applicable, the effect size with a confidence interval.	Section 2.

The Recommended Set

These items complement the Essential 10 and add important context to the study. Reporting the items in both sets represents best practice.

Item		Recommendation	Section/line number, or reason for not reporting
**Abstract**	11	Provide an accurate summary of the research objectives, animal species, strain and sex, key methods, principal findings, and study conclusions.	Abstract
**Background**	12	a. Include sufficient scientific background to understand the rationale and context for the study, and explain the experimental approach.	Section 1.
		b. Explain how the animal species and model used address the scientific objectives and, where appropriate, the relevance to human biology.	Section 1.
**Objectives**	13	Clearly describe the research question, research objectives and, where appropriate, specific hypotheses being tested.	Section 4.1
**Ethical statement**	14	Provide the name of the ethical review committee or equivalent that has approved the use of animals in this study, and any relevant licence or protocol numbers (if applicable). If ethical approval was not sought or granted, provide a justification.	Ethics Statement
**Housing and husbandry**	15	Provide details of housing and husbandry conditions, including any environmental enrichment.	Section 4.
**Animal care and monitoring**	16	a. Describe any interventions or steps taken in the experimental protocols to reduce pain, suffering and distress.	Section 4.4
		b. Report any expected or unexpected adverse events.	Section 4.4
		c. Describe the humane endpoints established for the study, the signs that were monitored and the frequency of monitoring. If the study did not have humane endpoints, state this.	Section 4.4
**Interpretation/ scientific implications**	17	a. Interpret the results, taking into account the study objectives and hypotheses, current theory and other relevant studies in the literature.	Section 2.
		b. Comment on the study limitations including potential sources of bias, limitations of the animal model, and imprecision associated with the results.	Section 3.
**Generalisability/ translation**	18	Comment on whether, and how, the findings of this study are likely to generalise to other species or experimental conditions, including any relevance to human biology (where appropriate).	Section 3.
**Protocol registration**	19	Provide a statement indicating whether a protocol (including the research question, key design features, and analysis plan) was prepared before the study, and if and where this protocol was registered.	Ethics Statement
**Data access**	20	Provide a statement describing if and where study data are available.	Data Avaliable Statement
**Declaration of interests**	21	a. Declare any potential conflicts of interest, including financial and non-financial. If none exist, this should be stated.	Competing Interests
		b. List all funding sources (including grant identifier) and the role of the funder(s) in the design, analysis and reporting of the study.	Funding
